# The impact of alcoholic drinks and dietary factors on epigenetic markers associated with triglyceride levels

**DOI:** 10.3389/fgene.2023.1117778

**Published:** 2023-02-15

**Authors:** Chao-Qiang Lai, Laurence D. Parnell, Yu-Chi Lee, Haihan Zeng, Caren E. Smith, Nicola M. McKeown, Donna K. Arnett, José M. Ordovás

**Affiliations:** ^1^ USDA ARS, Nutrition and Genomics Laboratory, JM-USDA Human Nutrition Research Center on Aging at Tufts University, Boston, MA, United States; ^2^ Nutrition and Genomics Laboratory, JM-USDA Human Nutrition Research Center on Aging at Tufts University, Boston, MA, United States; ^3^ Programs of Nutrition, Department of Health Sciences, Sargent College of Health and Rehabilitation Sciences, Boston University, Boston, MA, United States; ^4^ Nutrition Epidemiology and Data Science Friedman School of Nutrition Science and Policy, Tufts University, Boston, MA, United States; ^5^ Office of the Provost, University of South Carolina, Columbia, SC, United States; ^6^ IMDEA Food Institute, CEI UAM + CSIC, Madrid, Spain

**Keywords:** epigenetic mapping, DNA methylation, diet, lifestyle, triglyceride, cardiometabolic disease

## Abstract

**Background:** Many epigenetic loci have been associated with plasma triglyceride (TG) levels, but epigenetic connections between those loci and dietary exposures are largely unknown. This study aimed to characterize the epigenetic links between diet, lifestyle, and TG.

**Methods:** We first conducted an epigenome-wide association study (EWAS) for TG in the Framingham Heart Study Offspring population (FHS, *n* = 2,264). We then examined relationships between dietary and lifestyle-related variables, collected four times in 13 years, and differential DNA methylation sites (DMSs) associated with the last TG measures. Third, we conducted a mediation analysis to evaluate the causal relationships between diet-related variables and TG. Finally, we replicated three steps to validate identified DMSs associated with alcohol and carbohydrate intake in the Genetics of Lipid-Lowering Drugs and Diet Network (GOLDN) study (*n* = 993).

**Results:** In the FHS, the EWAS revealed 28 TG-associated DMSs at 19 gene regions. We identified 102 unique associations between these DMSs and one or more dietary and lifestyle-related variables. Alcohol and carbohydrate intake showed the most significant and consistent associations with 11 TG-associated DMSs. Mediation analyses demonstrated that alcohol and carbohydrate intake independently affect TG *via* DMSs as mediators. Higher alcohol intake was associated with lower methylation at seven DMSs and higher TG. In contrast, increased carbohydrate intake was associated with higher DNA methylation at two DMSs (*CPT1A* and *SLC7A11*) and lower TG. Validation in the GOLDN further supports the findings.

**Conclusion:** Our findings imply that TG-associated DMSs reflect dietary intakes, particularly alcoholic drinks, which could affect the current cardiometabolic risk *via* epigenetic changes. This study illustrates a new method to map epigenetic signatures of environmental factors for disease risk. Identification of epigenetic markers of dietary intake can provide insight into an individual’s risk of cardiovascular disease and support the application of precision nutrition.

**Clinical Trial Registration:** www.ClinicalTrials.gov, the Framingham Heart Study (FHS), NCT00005121; the Genetics of Lipid Lowering Drugs and Diet Network (GOLDN), NCT01023750.

## Introduction

Diet and lifestyle habits affect human health. As environmental exposures to the human genome are consistent and habitual, dietary intake and lifestyle behaviors modify epigenetic status without changing the genomic DNA sequence, but do affect the gene expression and the physiological function of cells and organelles (Aguilera et al., 2010; Cavalli and Heard, 2019). In turn, an altered physiology contributes to the risk of human diseases (Cavalli and Heard, 2019). However, the mechanisms underlying the alteration of epigenetic status through diet and lifestyle exposures are incompletely characterized. Increasing evidence supports that DNA methylation measurements from the peripheral blood mononuclear cell (PBMC) DNA are robust and relevant biomarkers of health status, as supported by the strong correlation between the methylation age and chronological age (Horvath, 2013; Issa, 2014). The DNA methylation age, measured in PBMCs, combined with plasma biomarkers, can accurately predict the biological age (Lu et al., 2019; McCrory et al., 2021). Furthermore, biological aging measured in PBMCs is associated with the diet and lifestyle habits (Quach et al., 2017). Thus, epigenetic marks measured from the PBMC DNA reflect biological aging and health status.

Epigenome-wide association studies have identified many epigenetic marks associated with metabolic and cardiovascular diseases (CVD) (van Dijk et al., 2015; Lai et al., 2016; Ling and Ronn, 2019; Samblas et al., 2019). The epigenetic markers associated with disease risk exhibit altered methylation profiles as a result of specific environmental factors that induced those epigenetic changes (Lai et al., 2018; Lai et al., 2020). Plasma triglyceride (TG) is a causal metabolic risk factor of CVD independent of other risk factors, including low-density cholesterol (LDL-C) (Raposeiras-Roubin et al., 2021). Importantly, elevated TG levels respond to the specific dietary and pharmacological intervention (Hunter and Hegele, 2017; Mason et al., 2020). In this study, our objective was to map epigenetic marks of diet and lifestyle habits for TG in two populations. To achieve this, we first identified epigenetic marks associated with TG and then examined the correlation between identified TG-associated epigenetic marks and diet and lifestyle factors longitudinally measured at four time points for up to 13 years in the Framingham Heart Study (FHS), followed by confirmation in the second population.

## Materials and methods

### The Framingham Heart Study

The Framingham Heart Study, launched in 1948, is a community-based longitudinal population study that recruited participants who self-identified as being of European descent and lived in Framingham, MA (Dawber et al., 1951). In 1971, the original FHS participants’ children and spouses were recruited to establish the Framingham Offspring Study (FHS) (Kannel et al., 1979). Participants of FHS were interviewed and clinically examined about every 4–8 years after that. In this study, we used data from participants who took part in one or more of the four examination cycles: exam 5 (1991–1995), exam 6 (1995–1998), exam 7 (1998–2001), and exam 8 (2005–2008) over a mean of 13 years. Only participants who completed the diet and health assessment questionnaires and for whom a whole-genome DNA methylation profile was available were included in this study. The age of participants at exam 8 ranged from 40 to 90 years with a mean of 64.7 years. These data were requested *via* controlled access from dbGaP (https://dbgap.ncbi.nlm.nih.gov, with study accessions: phs000007.v28.p10 and phs000007.v25.p9; downloaded on 27 September 2017).

### The Genetics of Lipid-Lowering Drugs and Diet Network (GOLDN) study

The GOLDN study was a subset of the Family Heart Study funded by the NIH National Heart, Lung, and Blood Institute (Corella et al., 2007; Lai et al., 2007). The study recruited a total of 1,327 participants with ages ranging from 18 to 92 years, with a mean age of 48.7 years from two study centers: Minneapolis, MN, and Salt Lake City, UT. The main goals of the study were to identify genetic variants responsible for individual variation in responses to a high-fat meal after a 3-week intervention of fenofibrate, a triglyceride-lowering mediation (Lai et al., 2007). The Institutional Review Boards at Tufts University, the University of Minnesota, the University of Utah, and the University of Alabama at Birmingham (United States) approved the study protocol. The present study included 474 men and 519 women with a completed whole-genome profile of DNA methylation and dietary data at baseline (Corella et al., 2007). The GOLDN cohort, of European descent, shares a similar ancestry as FHS, and it was a young cohort with a broad spectrum of alcohol intake. This makes GOLDN an appropriate population to validate the findings from FHS. The data can be accessed from dbGaP (https://dbgap.ncbi.nlm.nih.gov) with study accessions: phs000741.v2.p1.

### Dietary intake and food grouping

In FHS, foods and nutrients were derived from the 126-item modified Willett semi-quantitative food frequency questionnaire (FFQ) in the fifth to eighth study examinations (1991–2008) (Wang et al., 2014). Dietary exposures were classified as follows: 1) daily absolute intake of nutrients/bioactives, including all macronutrients, fiber, vitamins, minerals, and bioactives (i.e., absolute intake). Macronutrients (i.e., fat, carbohydrate, and protein) were further expressed and analyzed as percentages of total energy intake; 2) individual food items (servings/week or servings/day) as captured by the FFQ (i.e., 129 food items); and 3) food groups, whereby individual food items were classified into 31 food groups. All dietary and lifestyle variables were summarized in Supplementary Table S2. Physical activity scores were estimated based on the Paffenbarger questionnaire of the Harvard Alumni Activity Survey (Lee and Paffenbarger, 1998). The physical activity was not available for exam 6. Other lifestyle exposures included alcohol intake (grams per day or number of days per week of alcohol drinking) and smoking (number of cigarettes per day). In GOLDN, dietary assessment was conducted using the Diet History Questionnaire (Corella et al., 2007; Lai et al., 2007) and dietary intake was estimated based on the Harvard University Food Composition Database, the USDA database, and the Minnesota Nutrient System (Dawber et al., 1951). These unique characteristics of dietary records at four time points and epigenomic profiling available at exam 8 in FHS provided a solid foundation to examine the connection between obesity-associated epigenome signals and diet and lifestyle habits on TG.

### Genome-wide DNA methylation

Genome-wide DNA methylation of isolated DNA samples in both cohorts (FHS and GOLDN) was measured using Infinium HumanMethylation450 K arrays (Illumina) as described (Absher et al., 2013; Marioni et al., 2015). For FHS, DNA methylation data were requested from dbGaP (accession: phs000724.v9.p13). For both FHS and GOLDN, quality control (QC) processing was applied to the raw IDAT files as described (Morris et al., 2014; Lai et al., 2018). To adjust for the heterogeneity of cell-type composition in the blood across samples, we calculated principal components (PCs) with β scores of all filtered autosomal DNA methylation sites (DMSs) using the PCA function implemented in SNP and VARIATION SUITE 8.9.0 (SVS 8.9.0, GoldenHelix Inc., Bozeman, MT, USA). The first five PCs were used as covariates to control for heterogeneity of different cell types in all analyses, which was well demonstrated in previous studies (Hidalgo et al., 2014; Irvin et al., 2014). After QC, 415,202 DMSs remained and were included in this study. Among them, 76.7% (of total passing QC) of the CpGs were annotated as genic, whereas 23.8% of CpGs across the genome can be considered intergenic. Annotation was based on the human genome build GRCh37/hg19.

### Transcription analysis of epigenetic variants

FHS transcriptome data were acquired from dbGaP under accession #phe00002.v6. Transcriptome profile was performed using the Affymetrix Human Exon 1.0 ST array on mRNA isolated from PBMC collected from the FHS Offspring Cohort participants after overnight fasting at exam 8 (McManus et al., 2017). The quality control and normalization of the raw gene expression data have been described (Katz et al., 2006; Joehanes et al., 2013). In this study, we obtained gene expression data for 572 participants who were not taking medication for hypertension, dyslipidemia, or diabetes to avoid interference of the medications on gene expression. Of the 19 identified TG-associated DMS regions, transcript data were sought for the gene harboring that DMS. For 13 transcripts, such data were available. To determine whether identified TG-associated DMSs were associated with corresponding mRNA expression in the PBMC, we examined the correlation between each identified DMS and gene expressions of the targeted gene with mixed linear models while controlling for age and sex, cell-type heterogeneity, and family relation.

### Epigenetic mapping of environmental factors for plasma triglyceride

#### Step 1—Epigenome-wide association study for TG

We conducted an epigenome-wide scan for TG using a mixed linear regression model to identify DMSs associated with TG. Log-10 transformed TG was modeled as the dependent variable and DMSs as predictors while controlling for sex and age at exam 8, cell-type heterogeneity, and family relationship as a random effect. The analysis was implemented in SVS 8.9.0. A Bonferroni test was applied to correct for multiple testing with epigenome-wide significance at 1.10E-07 (Lai et al., 2016). The total phenotypic variance of TG explained by identified epigenetic loci was estimated in participants not taking lipid-lowering medication using the multi-locus mixed model while controlling for sex, age, cell-type heterogeneity, and family relationship. Smoking and physical activities, as a part of the environmental factors, were not adjusted in this model.

#### Step 2—Association between TG-associated DMSs and dietary intake and lifestyle factors

To identify environmental factors associated with TG-associated DMSs, we conducted environment and epigenetic association analyses with all three categorizations of dietary exposure (Supplementary Table S2), and with lifestyle factors, measured in each of the four exams of FHS. For each DMS, the DNA methylation level was modeled as the dependent variable in a linear mixed model with each dietary intake and lifestyle factor as a predictor while controlling for sex, age at exam 8, cell-type heterogeneity, and family relationship as a random effect. As age is the key factor that was associated with DNA methylation measured at exam 8, the association models at exams 5, 6, and 7 were further controlled for age at exam 8. The analyses were conducted in two models: all participants (All) while controlling for lipid-lowering medication and in a sample of participants without lipid-lowering medication (No lipid med). These association tests were conducted and implemented in the SVS (v8.9.0).

For each DMS as a dependent variable, to correct for multiple testing, we estimated the total number of independent variables represented by all dietary intake and lifestyle factors using a correlation matrix method (Li and Ji, 2005). For each of the four exams, all dietary intake predictors were calculated and classified in a similar way (Wang et al., 2014), with the number of dietary variables ranging from 267 to 391 (Supplementary Table S2, this range across the four exams was mainly the result of different availability of data on nutrient/bioactives). In all cases, the estimated independent factors ranged from 153 to 170. Using Bonferroni adjustment for each DMS, we corrected for multiple testing with *p* = 0.05/170, = 0.0003.

#### Step 3—Mediation analysis

As alcohol and carbohydrate intake were the strongest and most consistent exposures associated with TG-associated DMSs across all four exams, mediation analysis was conducted only in participants not taking lipid-lowering drugs (to avoid potential interference of the medication) to identify causal relationships between exposures of alcohol and carbohydrate intake, and TG with DMSs as the mediators. Furthermore, to test if alcohol and carbohydrate intake at earlier exams had a causal effect on TG at exam 8 *via* epigenetic status (as mediators), mediation analysis was conducted with alcohol and carbohydrate intake at earlier exams (exams 5, 6, and 7) as exposures. The CAUSALMED procedure in SAS 9.4 (SAS, Cary, NC) was used for the mediation analysis (Robins and Greenland, 1992; Pearl, 2001). To be consistent, both alcohol and carbohydrate consumption, which were normalized to the total energy intake at each exam, were treated as the exposure variables, with TG at exam 8 as the outcome variable and 11 DMSs for alcohol intake and 10 DMSs for carbohydrate intake at exam 8 as mediators. In addition, as age, smoking, physical activity, BMI, and medications for hypertension and diabetes were important factors that were associated with DNA methylation, which was measured at exam 8, the association analyses at exams 5, 6, and 7 were further adjusted for age, smoking, physical activity, BMI, medications for hypertension and diabetes at exam 8. The significance threshold was adjusted for multiple tests using the Bonferroni correction at *p*-value = 0.0045 (0.05/11). The total, direct, and indirect effects were estimated *via* mediation analysis. The natural indirect effect (NIE) quantified the effect of alcohol consumption on TG mediated by the DMS, while the natural direct effect (NDE) quantified the residual effect not mediated by the DMS. The total effect is the sum of the direct and the indirect effects (Robins and Greenland, 1992; Lok and Bosch, 2021). Mediation analysis was conducted further for different types of alcoholic drinks (servings/week) in all four exams using identical models.

### Validation in GOLDN

For validation, we only focused on two key dietary factors identified as having the greatest impact on TG, alcohol, and carbohydrate intake (Supplementary Table S4). Hence, the replication was conducted only for 12 identified TG-associated DMSs that were associated with alcohol (11 DMSs) and carbohydrate (10 DMSs) intake. We replicated three steps of epigenetic mapping in the GOLDN cohort. Step 1: 12 DMSs were examined for association with plasma TG in a mixed linear model while controlling for sex, age, geographic location, family relationship, and cell-type heterogeneity. Step 2: alcohol and carbohydrate intake normalized to the total energy were associated with 12 DMSs with adjustment for potential confounding factors as in FHS. Step 3: mediation analysis was conducted the same way as in FHS while controlling for potential confounding factors. For validation, no correction for multiple testing was applied (i.e., statistical significance at *p* ≤ 0.05).

## Results

### Epigenome-wide association of plasma triglyceride

The demographic characteristics of the participants of the FHS cohort at exam 8 are provided in Supplementary Table S1. To identify DMSs associated with plasma TG, we conducted an epigenome-wide association study (EWAS) while controlling for sex, age, BMI, family relationship, and cell-type heterogeneity. In the FHS cohort of 2,264 participants available at exam 8, we identified 28 DMSs at 16 genic regions significantly associated with plasma TG at the epigenome-wide significance of *p* ≤ 1.1E-07 (*n* = 2,264, Table 1). Considering the potential confounding effect of lipid-lowering medication on TG, we additionally conducted an EWAS in only those 1,184 participants not using lipid-lowering medication (Table 1). Only 10 loci associations reached epigenome-wide significance in the smaller sample (Table 1). Among the 28 identified loci, individual DMSs accounted for variance in TG ranging from 1.3% to 6.8%. In total, 28 loci accounted for 15.3% of TG phenotypic variance. As some loci were highly correlated, 19 loci were selected to represent the 28 loci based on clusters in the correlation matrix. Together, these 19 loci account for 15% of TG phenotypic variance.

**TABLE 1:** Epigenetic variants associated with fasting plasma triglyceride of the Framingham Heart Study at Exam 8.

						All participants (*n* = 2,264)^a^				Participants no lipid med (*n* = 1,184)^b^			
DMS	Chr	Position	Gene	^c^Genic region	^d^CpG island	*p*-value	Beta	Beta SE	Variance explained (%)	*p*-value	Beta	Beta SE	Variance explained (%)
cg17901584	1	55,353,706	*DHCR24*	TSS1500	S_Shore	**3.29E-15**	−0.598	0.075	2.7	**5.58E-10**	−0.657	0.105	3.1
cg03725309	1	109,757,585	*SARS*	genic	S_Shore	**5.12E-09**	−0.697	0.119	1.5	5.65E-06	−0.695	0.153	1.7
cg16246545	1	120,255,941	*PHGDH*	genic	S_Shore	**6.13E-12**	−0.520	0.075	2.1	9.75E-07	−0.508	0.103	2.0
cg14476101	1	120,255,992	*PHGDH*	genic	S_Shore	**7.05E-15**	−0.445	0.057	2.7	1.17E-07	−0.420	0.079	2.3
cg19693031	1	145,441,552	*TXNIP*	3′UTR		**1.52E-16**	−0.576	0.069	3.0	7.64E-06	−0.440	0.098	1.6
cg06690548	4	139,162,808	*SLC7A11*	genic		**1.38E-11**	−0.371	0.055	2.0	**2.57E-09**	−0.514	0.086	2.9
cg19494588	5	146,195,103	*PPP2R2B*	genic		**6.67E-10**	−0.371	0.060	1.7	1.00E-05	−0.351	0.079	1.6
cg26403843	5	158,634,085	*RNF145*	genic	N_Shelf	**9.61E-09**	0.393	0.068	1.5	5.27E-05	0.378	0.093	1.3
cg21429551	7	30,635,762	*GARS*	genic	S_Shore	**4.09E-11**	−0.366	0.055	1.9	**1.01E-09**	−0.466	0.076	3.0
cg03068497	7	30,635,838	*GARS*	genic	S_Shore	**7.89E-09**	−0.297	0.051	1.5	**7.61E-09**	−0.411	0.071	2.7
cg19390658	7	30,636,176	*GARS*	genic	S_Shore	**4.52E-10**	−0.415	0.066	1.7	**1.19E-11**	−0.603	0.088	3.7
cg05014727	10	6,214,016	*PFKFB3*	genic		**3.32E-08**	−0.474	0.086	1.3	4.16E-03	−0.321	0.112	0.7
cg26262157	10	6,214,079	*PFKFB3*	genic		**8.12E-08**	−0.470	0.087	1.3	5.51E-05	−0.455	0.112	1.3
cg07504977	10	102,131,012	intergenic	intergenic	N_Shelf	**2.04E-12**	0.608	0.086	2.2	2.64E-05	0.503	0.119	1.4
cg11376147	11	57,261,198	*SLC43A1*	genic		**8.43E-13**	−1.440	0.200	2.3	**2.21E-08**	−1.494	0.265	2.5
cg00574958	11	68,607,622	*CPT1A*	5′UTR	N_Shore	**3.98E-25**	−2.561	0.244	4.7	**9.43E-16**	−2.875	0.353	5.2
cg09737197	11	68,607,675	*CPT1A*	5′UTR	N_Shore	**8.55E-11**	−1.034	0.158	1.9	2.04E-05	−0.906	0.212	1.5
cg17058475	11	68,607,737	*CPT1A*	5′UTR	N_Shore	**3.81E-23**	−1.796	0.179	4.3	**1.86E-10**	−1.507	0.234	3.3
cg27431877	12	124,911,924	*NCOR2*	genic	S_Shore	**1.04E-07**	0.834	0.156	1.3	2.17E-02	0.481	0.209	0.4
cg07434438	16	72,961,899	*ZFHX3*	genic		**4.20E-08**	−0.599	0.109	1.3	1.42E-03	−0.466	0.146	0.8
cg20544516	17	17,717,183	*SREBF1;MIR33B*	genic	S_Shore	**7.85E-11**	0.982	0.150	1.9	7.57E-05	0.774	0.195	1.3
cg08129017	17	17,728,660	*SREBF1*	genic	S_Shore	**3.11E-09**	0.576	0.097	1.5	1.72E-05	0.547	0.127	1.5
cg11024682	17	17,730,094	*SREBF1*	genic	S_Shelf	**1.27E-09**	0.682	0.112	1.6	7.39E-03	0.393	0.146	0.6
cg22304262	19	47,287,778	*SLC1A5*	genic, 5′UTR	N_Shelf	**1.37E-08**	−0.499	0.088	1.4	2.72E-05	−0.500	0.119	1.4
cg02316713	21	43,619,559	*ABCG1*	TSS1500		**1.52E-08**	0.609	0.107	1.4	2.16E-04	0.538	0.145	1.1
cg27243685	21	43,642,366	*ABCG1*	genic	S_Shelf	**2.51E-14**	0.925	0.121	2.6	**3.24E-09**	0.949	0.159	2.8
cg00222799	21	43,655,464	*ABCG1*	genic	Island	**1.72E-10**	0.588	0.092	1.8	6.01E-04	0.423	0.123	1.0
cg06500161	21	43,656,587	*ABCG1*	genic	S_Shore	**1.23E-35**	1.355	0.107	6.7	**7.79E-14**	1.071	0.142	4.5

^a^ All participants adjusted for age, sex, family relationship, cell heterogeneity, and medications for lipid lowering, hypertension, diabetes.

^b^ Participants not using lipid-lowering medication adjusted for age, sex, family relationship, cell heterogeneity, and medication for hypertension and diabetes.

^c^ Genic region includes the transcription start site to the end of 3′UTR.

^d^ CpG Island indicates the location relative to CpG island.

### Dietary and lifestyle factors associated with DMS in four exams

To characterize and map epigenetic status in relation to environmental exposures, we first examined the association between 19 DMSs and all dietary measures and lifestyle factors in four exams over an average of a 13-year timeframe. For each TG-associated DMS, we examined its association with each of the dietary and lifestyle exposures in two linear mixed models: all participants (All) and participants not taking lipid-lowering medication (No lipid med), while controlling for sex, age at exam 8, family relationship, cell-type heterogeneity, and medications for hypertension and type 2 diabetes in four exams.

**FIGURE 1:**
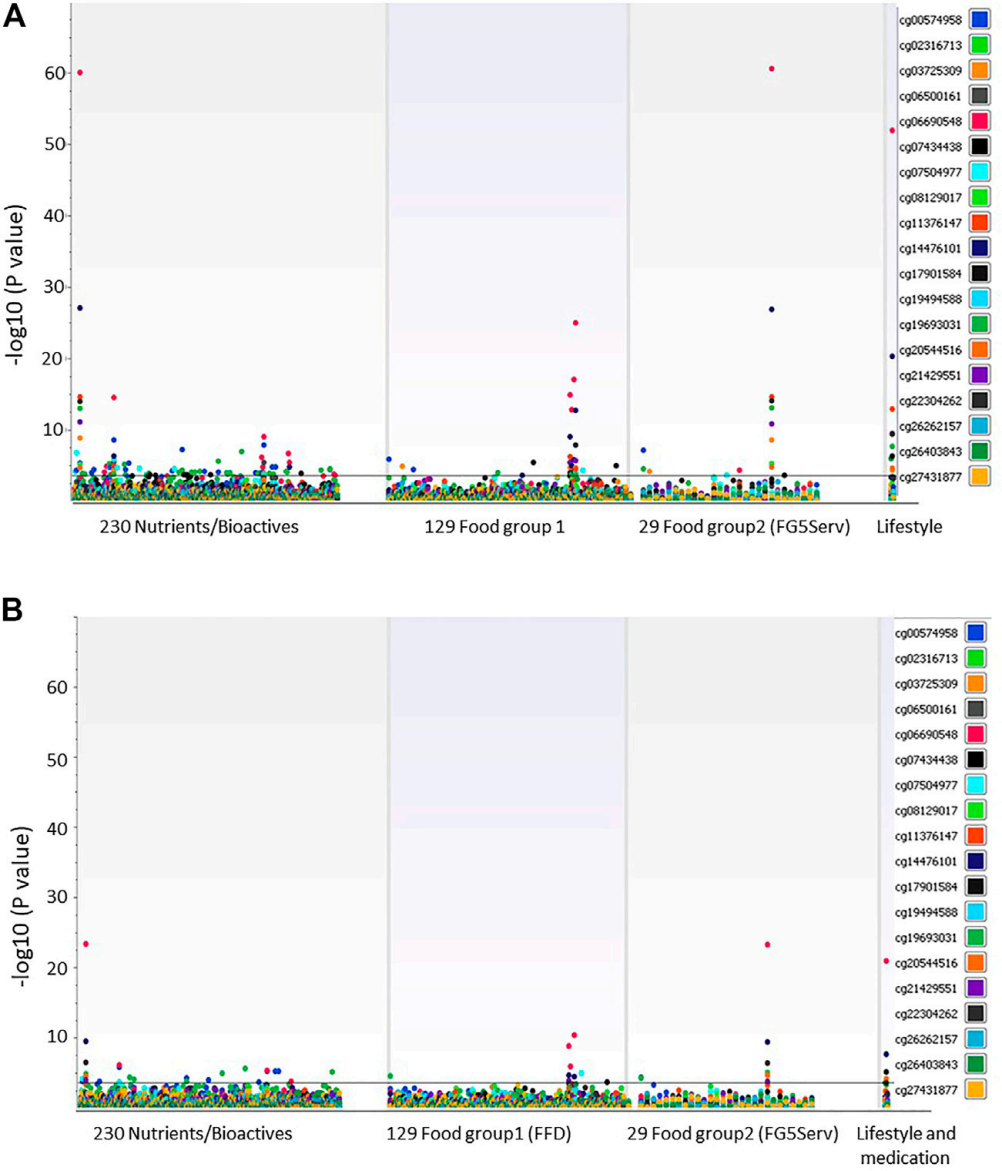
**(A)** Association between 19 DMSs and dietary intake and lifestyle factors at exam 8. All participants (*n* = 1919) were examined while controlling the association tests for sex, age at exam 8, cell-type, family relationship, and medications for lipid lowering, hypertension, and diabetes. **(B)** Association between 19 DMSs and dietary intake and lifestyle factors at exam 8. Association tests included participants (*n* = 1,041) not taking lipid-lowering medication while controlling for sex, age at exam 8, cell-type, family relationship, and medications for hypertension and diabetes.

Figure 1 shows the Manhattan plot of associations between 19 DMSs and all dietary and lifestyle factors measured and estimated at exam 8 for all participants (Figure 1A, *n* = 1923) and participants not taking lipid-lowering medication (Figure 1B, *n* = 1,041). After correction for multiple testing (*p* = 0.0003), 35 dietary and lifestyle variables were associated with methylation at cg06690548 in *SLC7A11* when all participants were included in the analysis (Figure 1A; Table 2). Only 10 of those dietary measures were associated with cg06690548 in participants who did not take the lipid-lowering medication (Figure 1B, *n* = 1,041).

**TABLE 2:** Numbers of dietary and lifestyle measures that were associated with each of 19 TG-associated epigenetic variants in four exams of the Framingham Heart Study.

DMS	Exam 5		Exam 6		Exam 7		Exam 8			
	All (*n* = 1800)	No lipid med (*n* = 1710)	All (*n* = 1985)	No lipid med (*n* = 1,746)	All (*N* = 2,014)	No lipid med (*N* = 1,618)	All (*N* = 1,923)	No lipid med (*N* = 1,041)	Total	Total—No lipid med
cg17901584	0	0	5	5	3	5	10	1	18	11
cg03725309	9	9	23	18	8	5	9	0	49	32
cg14476101	7	8	10	8	9	7	10	5	36	28
cg19693031	4	3	4	3	10	11	35	10	53	27
cg06690548	15	12	14	13	22	18	19	9	70	52
cg19494588	0	0	0	1	0	0	0	0	0	1
cg26403843	1	1	0	0	1	1	1	1	3	3
cg21429551	8	8	8	6	9	8	5	3	30	25
cg26262157	0	0	0	0	0	0	1	0	1	0
cg07504977	0	1	2	1	5	1	11	3	18	6
cg11376147	6	7	7	6	9	9	7	0	29	22
cg00574958	10	9	5	6	17	8	23	10	55	33
cg27431877	0	0	0	0	0	0	0	0	0	0
cg07434438	0	0	0	0	0	0	2	1	2	1
cg20544516	0	0	2	2	3	3	3	3	8	8
cg08129017	7	8	4	4	5	2	2	0	18	14
cg22304262	7	7	6	6	7	4	6	3	26	20
cg02316713	0	0	0	0	1	0	0	0	1	0
cg06500161	1	3	1	1	4	2	4	0	10	6
Total	75	76	91	80	113	84	148	49	427	289

In exam 8 with all participants (All), 16 of 19 DMSs were associated with at least one of the dietary and lifestyle variables (Table 2). Three DMSs (cg19494588, cg27431877, and cg02316713) showed few associations with dietary variables, and these could be more sensitive to exposures not analyzed here or not available in this population. Interestingly, across four exams, each DMS showed a similar pattern of association with dietary intake and lifestyle factors (Supplementary Figures S1A, B for exam 5, Supplementary Figures S2A, B for exam 6, and Supplementary Figures S3A, B for exam 7), with exam 8 showing the greatest number of significant associations. A summary of all significant associations between each DMS and each dietary and lifestyle variable is presented in two parts (Supplementary Table S3): in all participants (All) and in the subset limited to those not taking lipid medication (No lipid med). We observed 427 associations in all participants and 289 in participants not taking lipid-lowering medication summed over four exams, respectively, representing 102 and 74 unique associations between TG-associated DMSs and diet and lifestyle factors (Table 2, Supplementary Table S3). Among those associations between DMSs and dietary measures, we found exams 5, 6, and 7 shared, respectively, 37.2%, 37.2%, and 54.1% of the exam 8 associations for all participants, and 51.0%, 46.9%, and 67.3% for participants not taking lipid medication (Table 2).

To define the impact of specific diet and lifestyle habits as exposures that alter epigenetic status, we then ranked dietary and lifestyle measures by summarizing the total number of associations with DMSs over four exams (Supplementary Table S4). Among all dietary and lifestyle measures, the following dietary measures showed strong associations with 19 DMSs: alcohol intake (g/d), carbohydrate intake (% total energy intake), total sugar intake (g/d), smoking (number of cigarettes per day), vitamins B1 and B2 without counting supplements, dairy desserts/ice cream, calcium, animal fat/saturated fat, fat intake, vitamin D, and protein intake, accounting for 84.8% of all associations (Supplementary Tables S3, S4).

A total of 11 DMSs were most strongly associated with alcohol intake across all four exams with *p*-values varying from 2.89E-04 to 8.37E-70, with individual DMSs accounting for methylation variation ranging from 0.7% to 14.6% (Table 3). Interestingly, among the 10 DMSs that were associated with carbohydrate intake (% total energy intake), nine also were associated with alcohol intake, yet these nine DMS were associated with alcohol and carbohydrate intake in opposite directions (Table 3).

**TABLE 3:** 12 Epigenetic variants associated with alcohol consumption or/and carbohydrate intake across four exams in FHS.

Exam	DMS	Alcohol intake (grams/day)				Carbohydrate intake (% total energy)			
		*p*-value	Beta	Beta SE	Variance explained (%)	*p*-value	Beta	Beta SE	Variance explained (%)
5	cg03725309	**5.10E-11**	−0.00037	0.00006	2.4	0.004	0.00030	0.00010	0.5
	cg14476101	**7.43E-23**	−0.00112	0.00011	5.3	**3.01E-08**	0.00116	0.00021	1.7
	cg19693031	**1.13E-07**	−0.00050	0.00009	1.6	0.054	0.00033	0.00017	0.2
	cg06690548	**2.10E-56**	−0.00188	0.00011	13.1	**2.92E-18**	0.00193	0.00022	4.2
	cg21429551	**1.09E-19**	−0.00109	0.00012	4.5	**1.87E-06**	0.00105	0.00022	1.3
	cg07504977	0.024	0.00018	0.00008	0.3	1.27E-03	−0.00046	0.00014	0.6
	cg11376147	**7.61E-13**	−0.00024	0.00003	2.8	**8.37E-07**	0.00030	0.00006	1.4
	cg00574958	**5.19E-06**	−0.00013	0.00003	1.2	**1.47E-05**	0.00022	0.00005	1.0
	cg20544516	0.009	0.00012	0.00004	0.4	0.334	−0.00008	0.00008	0.1
	cg08129017	**1.09E-08**	0.00039	0.00007	1.8	0.045	−0.00025	0.00012	0.2
	cg22304262	**8.08E-16**	−0.00058	0.00007	3.6	**1.35E-05**	0.00058	0.00013	1.1
	cg06500161	0.034	0.00013	0.00006	0.3	0.004	−0.00032	0.00011	0.5
6	cg03725309	**5.25E-11**	−0.00036	0.00005	2.2	1.89E-03	0.00030	0.00010	0.5
	cg14476101	**3.93E-29**	−0.00126	0.00011	6.2	**5.11E-09**	0.00116	0.00020	1.7
	cg19693031	**7.14E-13**	−0.00066	0.00009	2.6	**3.03E-04**	0.00058	0.00016	0.7
	cg06690548	**8.37E-70**	−0.00205	0.00011	14.6	**9.30E-21**	0.00195	0.00021	4.3
	cg21429551	**1.02E-19**	−0.00107	0.00012	4.1	**3.55E-05**	0.00085	0.00021	0.9
	cg07504977	0.007	0.00021	0.00008	0.4	5.24E-04	−0.00047	0.00013	0.6
	cg11376147	**9.84E-16**	−0.00026	0.00003	3.2	**4.68E-07**	0.00029	0.00006	1.3
	cg00574958	**2.89E-04**	−0.00010	0.00003	0.7	**3.03E-07**	0.00024	0.00005	1.3
	cg20544516	**2.44E-05**	0.00018	0.00004	0.9	0.029	−0.00017	0.00008	0.2
	cg08129017	**3.64E-06**	0.00031	0.00007	1.1	0.002	−0.00037	0.00012	0.5
	cg22304262	**4.61E-14**	−0.00056	0.00007	2.8	2.12E-03	0.00040	0.00013	0.5
	cg06500161	1.26E-03	0.00019	0.00006	0.5	1.59E-02	−0.00025	0.00011	0.3
7	cg03725309	**1.22E-12**	−0.00038	0.00005	2.5	**5.82E-05**	0.00036	0.00009	0.8
	cg14476101	**2.89E-32**	−0.00131	0.00011	6.8	**2.47E-09**	0.00113	0.00019	1.8
	cg19693031	**5.43E-13**	−0.00066	0.00009	2.6	**1.25E-05**	0.00067	0.00015	0.9
	cg06690548	**4.69E-66**	−0.00195	0.00011	13.7	**8.00E-23**	0.00192	0.00019	4.7
	cg21429551	**8.43E-18**	−0.00100	0.00011	3.6	**3.46E-09**	0.00116	0.00019	1.7
	cg07504977	0.012	0.00019	0.00008	0.3	**3.23E-06**	−0.00059	0.00013	1.1
	cg11376147	**8.89E-16**	−0.00026	0.00003	3.2	**2.06E-07**	0.00028	0.00005	1.3
	cg00574958	**7.54E-06**	−0.00012	0.00003	1.0	**3.01E-11**	0.00030	0.00004	2.2
	cg20544516	**2.74E-05**	0.00018	0.00004	0.9	0.007	−0.00020	0.00007	0.4
	cg08129017	**1.05E-05**	0.00030	0.00007	1.0	0.088	−0.00019	0.00011	0.1
	cg22304262	**1.31E-11**	−0.00050	0.00007	2.3	**5.90E-05**	0.00050	0.00012	0.8
	cg06500161	**1.01E-04**	0.00023	0.00006	0.8	**1.06E-06**	−0.00048	0.00010	1.2
8	cg03725309	**1.68E-09**	−0.00031	0.00005	1.9	**4.71E-05**	0.00039	0.00009	0.9
	cg14476101	**1.01E-27**	−0.00118	0.00011	6.1	**5.43E-07**	0.00099	0.00020	1.3
	cg19693031	**1.17E-13**	−0.00065	0.00009	2.9	**1.85E-04**	0.00060	0.00016	0.7
	cg06690548	**8.39E-61**	−0.00183	0.00011	13.3	**3.20E-15**	0.00163	0.00021	3.2
	cg21429551	**9.17E-12**	−0.00077	0.00011	2.4	0.007	0.00055	0.00021	0.4
	cg07504977	0.047	0.00015	0.00007	0.2	**1.48E-06**	−0.00064	0.00013	1.2
	cg11376147	**2.74E-15**	−0.00025	0.00003	3.2	**1.23E-05**	0.00025	0.00006	1.0
	cg00574958	**5.12E-06**	−0.00012	0.00003	1.1	**3.13E-09**	0.00028	0.00005	1.8
	cg20544516	**2.15E-05**	0.00018	0.00004	0.9	0.002	−0.00023	0.00008	0.5
	cg08129017	**7.41E-06**	0.00029	0.00007	1.1	0.002	−0.00037	0.00012	0.5
	cg22304262	**1.06E-14**	−0.00055	0.00007	3.1	0.017	0.00031	0.00013	0.3
	cg06500161	0.008	0.00015	0.00006	0.4	**3.46E-06**	−0.00047	0.00010	1.1

### Mediation analysis: Effects of alcohol and carbohydrate intake on TG

**FIGURE 2:**
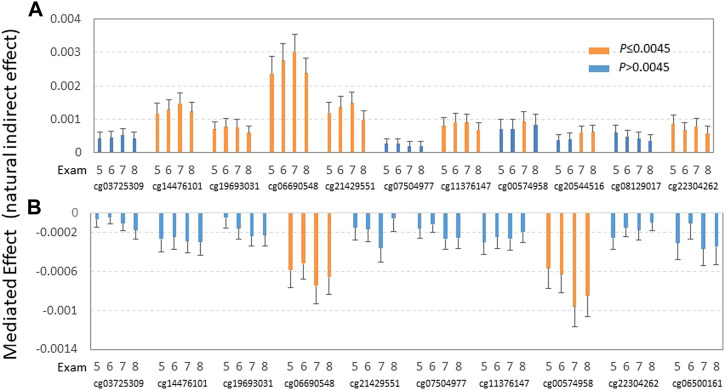
Effects of alcohol consumption **(A)** and carbohydrate intake **(B)** on plasma TG *via* epigenetic mediators. Mediation analysis was conducted in all four exams while controlling for sex, age, BMI, physical activity, smoking status, cell-type heterogeneity, and medications for type 2 diabetes and hypertension at exam 8 (as DNA methylation was measured at exam 8). **(A)** Indirect effects of alcohol intake (% total energy) on TG through 11 DMSs as mediators were estimated for four exams (exams 5—8) using mediation analysis in participants not taking lipid-lowering medication while controlling for covariates (sex, age BMI, physical activity, smoking status, cell-type heterogeneity, and medication for type 2 diabetes and hypertension at exam 8). **(B)** Indirect effects of carbohydrate intake (% total energy intake) on TG through 10 DMSs as mediators were estimated for four exams (exams 5–8) using mediation analysis in participants not taking lipid-lowering medication while controlling for covariates (sex, age, BMI, physical activity, smoking status, cell-type heterogeneity, and medication for type 2 diabetes and hypertension at exam 8). Orange and blue bars indicate significant and non-significant mediation effects of carbohydrate intake on TG after correction for multiple testing, respectively.

Considering the strong association between DMSs with TG and alcohol and carbohydrate consumption, and the opposing direction of the influence of these two dietary factors, we conducted a mediation analysis to examine the potential causal effects of alcohol and carbohydrate intake on plasma TG. Mediation analysis was conducted in all four exams only in those participants not taking lipid-lowering medication to exclude the effect of lipid medication on TG while controlling for covariates (sex, age, BMI, physical activity, smoking status, cell-type heterogeneity, and medication for type 2 diabetes and hypertension at exam 8). For exam 8, seven DMSs (cg14476101, cg19693031, cg06690548, cg21429551, cg11376147, cg20544516, and cg22304262) exhibited significant mediated effects related to alcohol intake (% total energy) on TG (Figure 2A, Supplementary Table S5). Notably, the positive direction of the estimated value remained the same for all these sites across all four exams, whereas the natural indirect effect (NIE) of cg20544516 became insignificant in exams 5 and 6. This suggests that the positive effects of alcohol intake on TG are mediated through seven DMSs at seven genes (*PHGDH*, *TXNIP*, *SLC7A11*, *GARS*, *SLC43A1*, *SREBF1*, and *SLC1A5*). Also, we found that *TXNIP*-cg19693031, *SLC7A11*-cg06690548, *GARS*-cg21429551, and *CPT1A-*cg00574958 showed a negative natural direct effect (NDE) on (decreased) TG in exams 7 and 8 (Supplementary Table S5).

To determine if different types of alcohol exhibit differential mediated effects on TG, we undertook further mediation analysis by four types of alcoholic drinks: beer, red wine, white wine, and liquor (all as servings per week). As shown in Supplementary Figure S4, beer and liquor showed strong indirect mediated effects (NIE) on (increased) TG through *SLC7A11*-cg06690548 over all four exams and to some extent, through cg14476101 and cg21429551 over most of the four exams. Red wine and white wine showed mediated significant positive effects on TG only *via SLC7A11*-cg06690548. Interestingly, as shown in Supplementary Figure S5, while not significant, red and white wine showed negative non-mediated effects (NDE—not through mediation) on (decreased) TG, whereas beer and liquor showed no trend for these effects on TG.

For carbohydrate intake, as a percentage of the total energy, only DMSs cg06690548 and cg00574958 showed significant negative mediation effects on (decreased) TG for all four exams (Figure 2B, Supplementary Table S6). This observation suggests that carbohydrate intake shows negative effects as TG through those DMSs at genes *ABCG1* and *CPT1A*. On the other hand, all 10 DMSs show significant positive non-mediated effects (natural direct effect) on TG in exam 8, but not in other exams, except for cg00574958 in exam 7. This observation implies that the non-mediated effects of carbohydrate are not as long-lasting as those mediated effects through DMS as mediators from carbohydrate consumption.

To determine if alcohol consumption and carbohydrate intake mediate effects on TG independently of each other, we performed the mediation analysis for carbohydrate and alcohol while controlling additionally for alcohol intake or carbohydrate intake, respectively. The results showed that both mediated effects remain significant after mutual adjustment (alcohol consumption or carbohydrate intake reciprocally, Supplementary Tables S5, S6), except for carbohydrate intake on TG at cg06690548 (*p* = 0.4283). This underscores that alcohol consumption and carbohydrate intake mostly affect TG independently through the mediators of the epigenetic status of the respective genes. In addition, there were no significant interactions between the mediators (DMSs) and exposures (alcohol or carbohydrate intake) effect on TG (Supplementary Tables S5, S6).

### Validation in GOLDN

The general characteristics of the participants of the GOLDN population are provided in Supplementary Table S1. Compared to the FHS cohort, the amount of alcohol consumption is significantly lower (2.0% vs. 10.8% of the total energy) in the GOLDN population. The participants of GOLDN are younger (mean age: 48.6 vs. 66.3) than those in the FHS at exam 8.

For validation of the 12 identified DMSs that were associated with alcohol (11 DMSs) and carbohydrate (10 DMSs) intake in FHS, we replicated the three steps of epigenetic mapping analysis previously performed in the GOLDN study. For Step 1, epigenetic associations of 12 DMSs and TG are presented in Supplementary Table S7. Except for cg20544516 and cg22304262, all DMSs showed significant association with TG. For Step 2, as shown in Supplementary Table S8, all DMSs, except for 3 (cg19693031, cg21429551, and cg07504977), exhibited significant association with alcohol intake. Five of 10 DMSs showed significant trends or associations with carbohydrate intake. For Step 3, mediation analysis (Supplementary Table S8), four of the DMSs showed the significant positive mediated effects of alcohol intake on TG, whereas three DMSs mediated negative effects of carbohydrate intake on TG. Considering the differences between GOLDN and FHS for mean age (48.7 years—GOLDN *vs*. 66.3 years–FHS) and geographic locations, it is evident that the results in two cohorts are similar, especially for the mediation analysis.

### Epigenetic variants and associated gene expression

To examine whether identified TG-associated DMSs are associated with the transcription activity, we examined the correlation between these DMSs and the expression of the genes to which the DMSs were mapped. This was done using a mixed linear regression model for the participants of the FHS cohort who did not use medications for hypertension, dyslipidemia, or diabetes and controlling for cell-type heterogeneity and age and sex. Among 19 DMSs, gene expression data for 13 corresponding genes were measured in the PBMC at exam 8. Eight of 13 DMSs were negatively associated with expression of the accompanying gene in which the DMS is located (Supplementary Table S9). This observation supports a function for epigenetic variants in regulating gene expression, such that DNA methylation generally suppresses the expression of the targeted gene (Jones, 2012). The non-significant association observed for the other five genes could be because of the low expression levels in PBMC or cyclical characteristics of the expression.

## Discussion

This study sought to characterize the nature of the relationship between the epigenetic status and diet and lifestyle for TG. To accomplish this, we first identified DMSs associated with TG by conducting an EWAS and then examined the relationship between those TG-associated DMSs and diet and lifestyle habits over a period of ∼13 years. While there was a trend for more factors associated with TG-epigenetic marks in the last exam than in the earlier exams, several dietary factors showed a consistent correlation with epigenetic marks over all four exams. The most impactful dietary and lifestyle factors include alcohol and carbohydrate intake, total sugar, smoking, vitamins B1 and B2, dairy desserts, calcium, saturated fat, total fat, vitamin D, protein, and sweet baked foods (Supplementary Table S3). The validation in a second cohort (GOLDN) further supports the consistent associations of alcohol and carbohydrate intake with TG-associated epigenetic marks.

TG is a causal risk factor for CVD (Raposeiras-Roubin et al., 2021), in addition to LDL-C. EWAS identified 19 independent DMSs, which accounted for ∼15% of the total TG variation. Over four exams, we observed many associations between the 19 TG-associated DMSs and diet and lifestyle factors, encompassing 102 of these factors. The strongest and most consistent associations were alcohol and carbohydrate intake, representing 11 of 19 DMSs. Alcohol intake accounts for 13.3% of cg06690548 methylation variation at *SLC7A11*. Although high alcohol intake (greater than two drinks/day) was associated with increased TG (Foerster et al., 2009; Klop et al., 2013), other studies have indicated that alcohol intake is associated with increased HDL-C and decreased TG, and increased risk of hypertension, coronary heart disease, and myocardial infarction (Rosoff et al., 2020). A recent study based on the UK Biobank cohort demonstrated that the apparent benefits of light or moderate alcohol intake was diminished by healthy lifestyle factors and, importantly, any amount of alcohol consumption was associated with an increased CVD risk (Biddinger et al., 2022). The lifetime average consumption of alcohol is positively associated with accelerated biological aging, as estimated by GrimAge (Kresovich et al., 2021) with cg06690548 at *SLC7A11* being the key contributor to GrimAge (Lu et al., 2019). Our study found that 13 of 19 DMSs were associated with alcohol intake. The mediation analysis results further support that the effects of alcohol intake increased TG *via* differential DNA methylation of seven DMSs at *PHGDH*, *TXNIP*, *SLC7A11*, *GARS*, *SLC43A1*, *SREBF1*, and *SLC1A5*. The different types of alcoholic drinks, notably beer, red wine, white wine, and liquor, showed consistent mediated effects on TG through CpG methylation at *SLC7A11*. Although total alcohol intake decreased population-wide from exam 5 to exam 8 (Parekh et al., 2021), all 11 DMSs exhibited consistent associations with alcohol intake across the four exams (Table 3). Alcohol could have a cumulative effect on DMSs from exam 5 to exam 8 (Table 3), but this remains to be unequivocally illustrated. The high consumption of alcohol affecting risk of CVD, myocardial infarction, and aging could be confounded by unhealthy lifestyle choices such as smoking. Nevertheless, our results suggest that alcohol and carbohydrate intakes and smoking are the most critical lifestyle factors acting epigenetically to modulate plasma TG.

Alcohol is more energy dense than carbohydrates. In this study, our results indicated that alcohol intake and carbohydrate intake exhibited opposite effects on TG through the epigenetic mechanisms. This can be explained as the epigenetic regulation of gene expression by dietary exposure. Alcohol intake was strongly and negatively associated with nine DMSs, except cg20544516 (i.e., a positive association), across four exams (Table 3: Supplementary Table S3). Considering that low methylation generally led to high expression of the targeted genes (Supplementary Table S9), high consumption of alcohol intake increased the gene expression, and increased TG, for example, cg06690548 at *SLC7A11* (Lohoff et al., 2021). Indeed, mediation analysis implied that alcohol intake was associated with increased TG through seven DMSs in seven gene regions (*PHGDH*, *TXNIP*, *SLC7A11*, *GARS1*, *SLC43A1*, *CPT1A*, and *SLC1A5*) as mediators. In contrast, carbohydrate intake was strongly correlated with six of the same DMSs (excluding *PHGDH*), but in the opposite direction (Table 3). High methylation generally leads to low expression of targeted genes. Thus, high intake of carbohydrate intake causes low expression of targeted genes (Supplementary Table S9), for example, cg00574958 at *CPT1A* (Lai et al., 2020), and decreased TG. Again, the mediation results support that carbohydrate shows negative effects on (decreased) TG through two DMSs (cg00574958 and cg06690548) as mediators. From the perception of biochemistry and metabolism, the opposite effects of alcohol and carbohydrate on TG reflected by their different effects on hepatic lipid metabolism. Alcohol can induce hypoglycemia (Marks, 1978) and disturb all aspects of hepatic lipid metabolism through fatty acid transporters, TG synthesis, and mitochondrial β-oxidation (You and Arteel, 2019). Among the seven genes identified by mediation analysis, *SCD* and *SREBF1* function in TG synthesis, and *CPT1A* and *TXNIP* are involved in mitochondrial β-oxidation. Alcohol directly and indirectly affects expression of these genes through epigenetic modification and then hepatic lipid flux which ultimately leads to lipid accumulation. In contrast, carbohydrates are essential nutrients for energy metabolism. There is strong evidence that carbohydrate intake, which accelerates the elimination of alcohol from the blood, can counteract this effect of alcohol intake (Rogers et al., 1987), thereby having the opposite effect on the epigenetic state. Thus, to some extent, carbohydrate intake counteracts the effects of alcohol and then has an opposite effect on TG through epigenetic modification of target genes, especially *SLC7A11* and *CPT1A*, although the exact molecular mechanism remains to be elucidated.

From a mechanistic perspective, male C57BL/6J mice fed an ethanol-containing diet exhibited higher levels of liver TG, indicating hepatic steatosis and, interestingly, altered diurnal oscillations of core clock genes in the liver but not in the suprachiasmatic nucleus, compared to control mice (Filiano et al., 2013). The prominent findings in the current analysis are the consistent associations between TG-associated DMSs and alcohol, with alcohol acting as the mediator to affect TG. Many of those same DMSs have been observed as associated with alcohol and diseases consequential to heavy drinking. For example, a recently published EWAS identified the same CpG sites noted here in *SLC7A11*, *SLC43A1*, and *PHGDH*, with a different CpG observed in *SLC1A5*, all associated with alcohol consumption (Lohoff et al., 2021). The top EWAS probe cg06690548, mapped to cystine/glutamate transporter *SLC7A11*, was replicated in the second cohort of alcohol use disorders (AUD) and control participants showing strong hypomethylation in AUD (*P* < 1E-17). Importantly, decreased methylation at cg06690548 in *SLC7A11* was consistently associated with clinical measures, including increased heavy drinking days. Additionally, hypomethylation at cg06690548 was associated with the elevated total cholesterol and TG levels (Lohoff et al., 2021). Regarding *PHGDH*, encoding phosphoglycerate dehydrogenase, increased lipid accumulation, and reduced NAD + activity were seen in mouse *Phgdh*-knockout primary hepatocytes incubated with free fatty acids, effects that were reversed upon *Phgdh* overexpression, including reduced hepatic TG accumulation (Sim et al., 2020). SLC1A5 is known as a transporter of alanine, serine, and cysteine but transports glutamine in a Na + -dependent manner in the liver (Scalise et al., 2018). A comparison of rats fed a high-alcohol diet either supplemented with glutamine (at 0.84%) or not indicated that hepatic fat deposition, inflammation, altered liver function, and hyperammonemia in the glutamine group were all attenuated (Xiao et al., 2021).

The dietary assessment of the FHS cohort from exams 5 to 8 in 13 years uses data from four standardized exams (Wang et al., 2014), making their use in such analyses as presented here a distinct advantage. This study examined all dietary intakes measured at four different time points in FHS. Several key foods, such as alcohol, carbohydrate, ice cream, and sugar, plus smoking, showed consistent correlation with the identified DMSs across four exams. In sum, our results clearly show that the observed associations between methylation levels at specific CpGs and outcomes related to metabolic diseases can be strongly mediated by various exposures. Hence, we believe that this epigenetic mapping approach can be applied to other environmental exposures in relation to any phenotype and disease. While many environmental factors, such as diet, pollutant exposure, and social stress, are known to be associated with disease risk, it is often speculated under what mechanism and if such exposures are the cause of the disease risk. Our approach illustrated that if an environmental factor is a potential cause of disease through epigenetic mechanisms, then the identified epigenetic markers can be used for prediction of cardiovascular disease, for example, with prediction based on machine learning methods (Lee et al., 2022). Hence, identification and understanding of the epigenetic markers signifying exposures may facilitate diagnostics and prevention of such diseases by modifying those exposures.

This study is not without its limitations. One of those is the measurements of epigenetic status were performed in PBMCs, which may not be the optimal tissue for epigenetic signals of diet and lifestyle habits as related to TG. Yet, DNA methylation measured from blood DNA can accurately predict the biological age (Horvath, 2013), which is associated with environmental exposure (Quach et al., 2017). Second, the loci described here are from the study population alone, and are not to be considered as general-use biomarkers of exposure to alcoholic drinks or other dietary factors, because equating the methylation status at specific loci with exposure to alcohol would be unethical (Santalo and Berdasco, 2022). In addition, although the associations between TG-associated DMSs at exam 8 and diet and lifestyle habits were observed in four exams in 13 years and confirmed by replication in a second cohort (GOLDN), it must be recognized that this evidence does not rule out the reverse causation of TG on DNA epigenetic changes (Davey Smith and Hemani, 2014; Sayols-Baixeras et al., 2018). Furthermore, such epigenetic marks of diet and lifestyle could be specific to given environments and populations. Therefore, the conclusions based on the findings from the current study must be interpreted with caution.

In conclusion, this study illustrated an example to map epigenetic signatures of diet and lifestyle habits for TG. Our results indicate that dietary factors of alcohol and carbohydrate are associated with specific DNA methylation markers and could mediate the observed associations between diet and cardiometabolic risk factors. Epigenetic markers of dietary intake provide insight into an individual’s risk of cardiovascular disease and improve the application of precision nutrition.

## Data Availability

The datasets presented in this article are not readily available because controlled access datasets were analyzed in this study. Requests to access the datasets should be directed to dbGaP (https://dbgap.ncbi.nlm.nih.gov) under the accession numbers phs000007.v25.p9, phs000007.v28.p10, phs000342.v18.p11, phs000724.v9.p13, phs000492.v2.

## Abbreviations

AUD, alcohol use disorders; CVD, cardiovascular disease; DMSs, DNA methylation sites; EWAS, epigenome-wide association study; LDL-C, low-density cholesterol; NDE, natural direct effect; NIE, natural indirect effect; PBMC, peripheral blood mononuclear cell; TG, plasma triglyceride.
