# Supplementary figures and images for: The impact of alcoholic drinks and dietary factors on epigenetic markers associated with triglyceride levels
Source: Front Genet. 2023 Feb 15;14:1117778. doi: 10.3389/fgene.2023.1117778 (PMC9975169; doi:10.3389/fgene.2023.1117778)

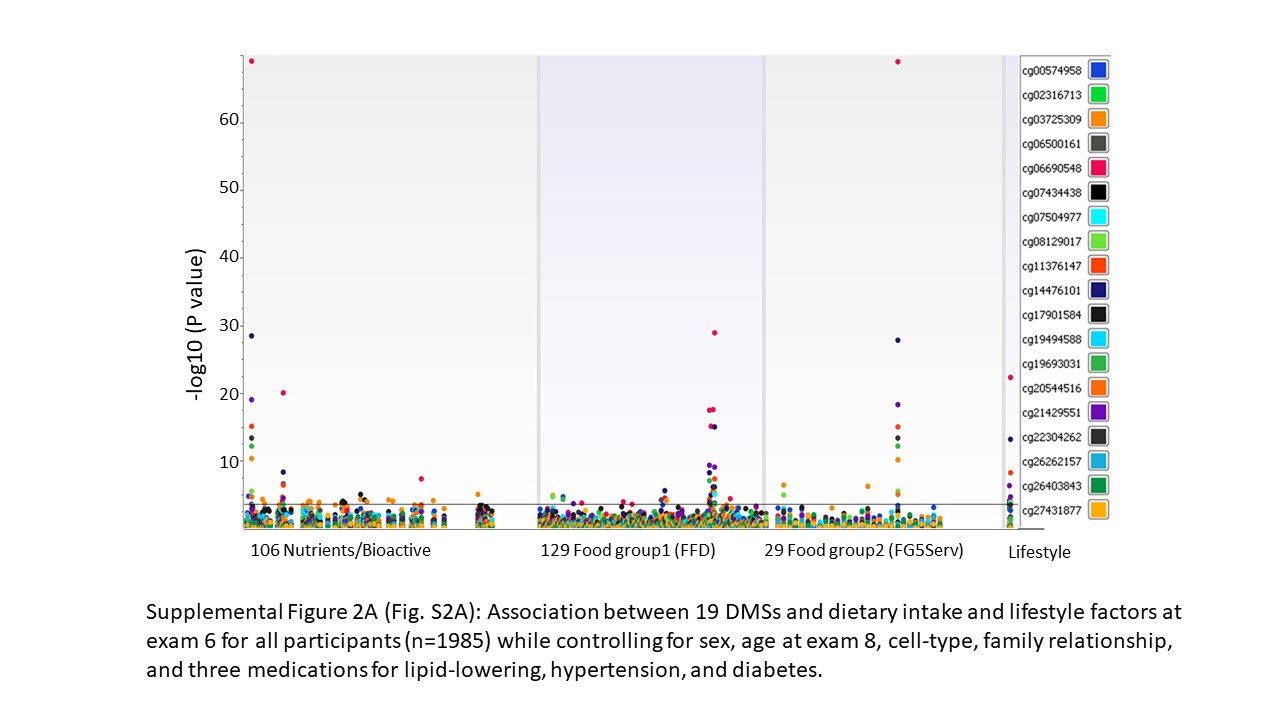

Supplement: Supplementary file 1 [file Image3.JPEG]

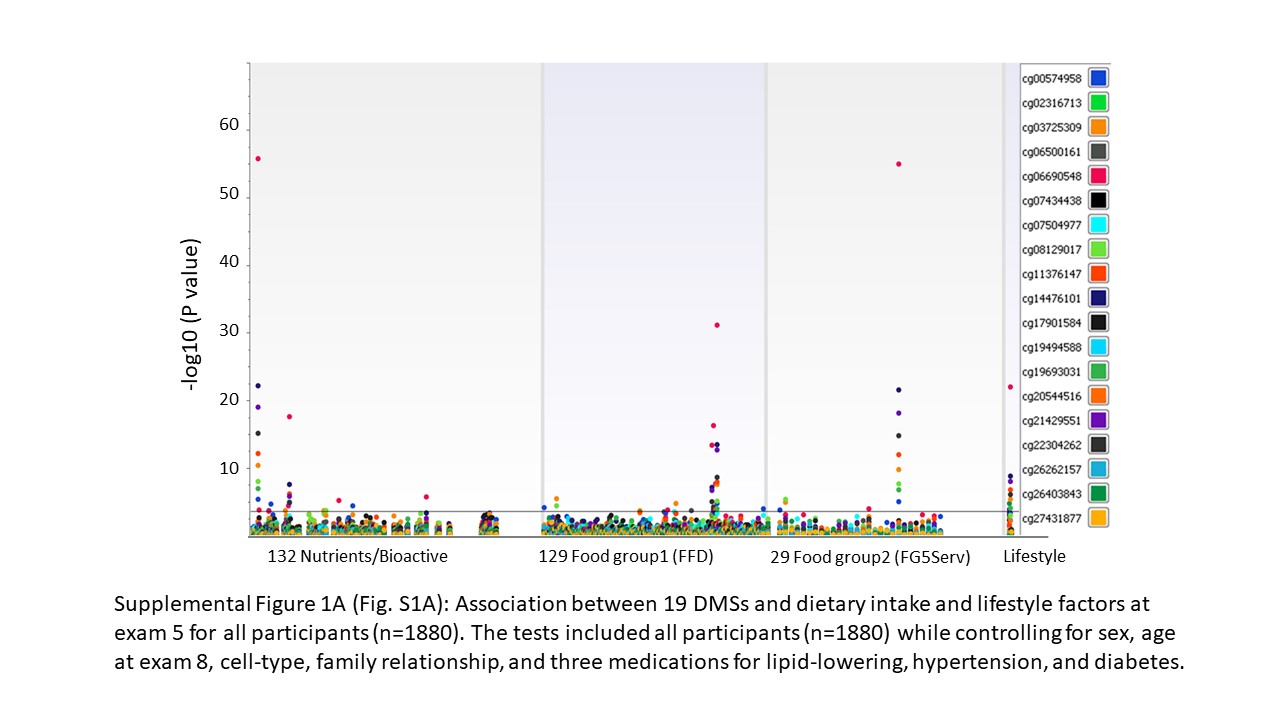

Supplement: Supplementary file 2 [file Image1.JPEG]

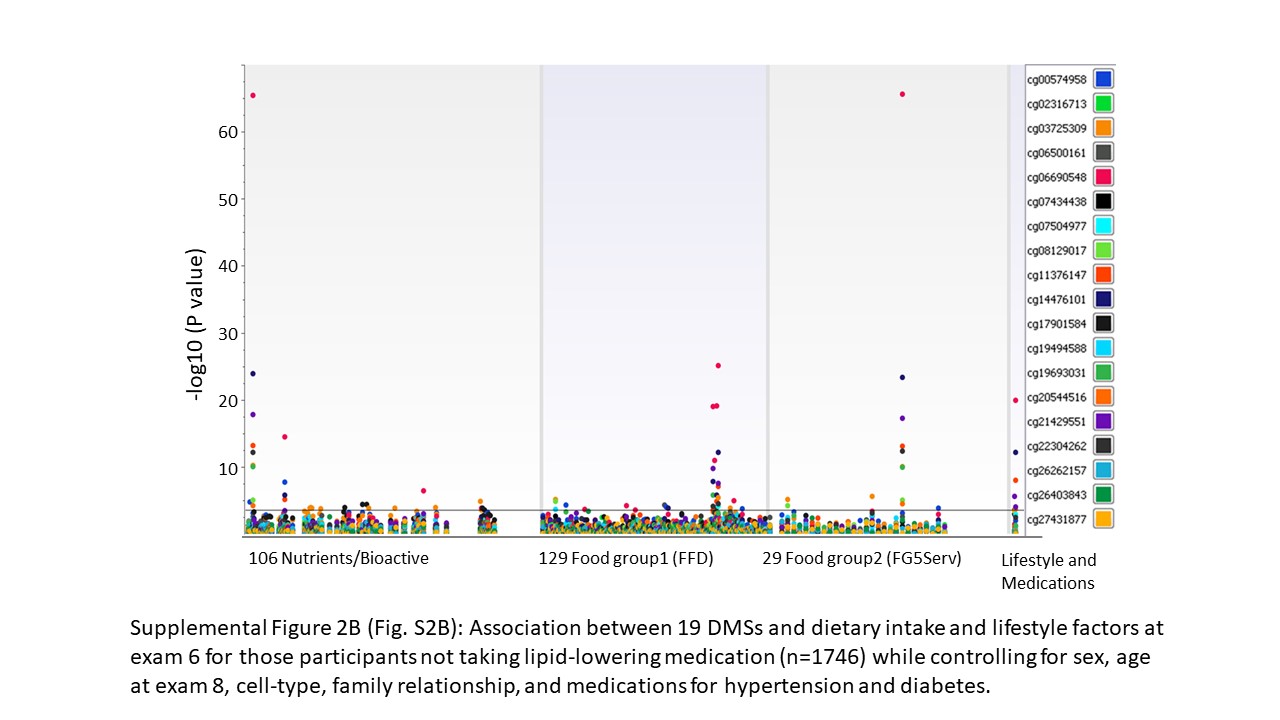

Supplement: Supplementary file 3 [file Image4.JPEG]

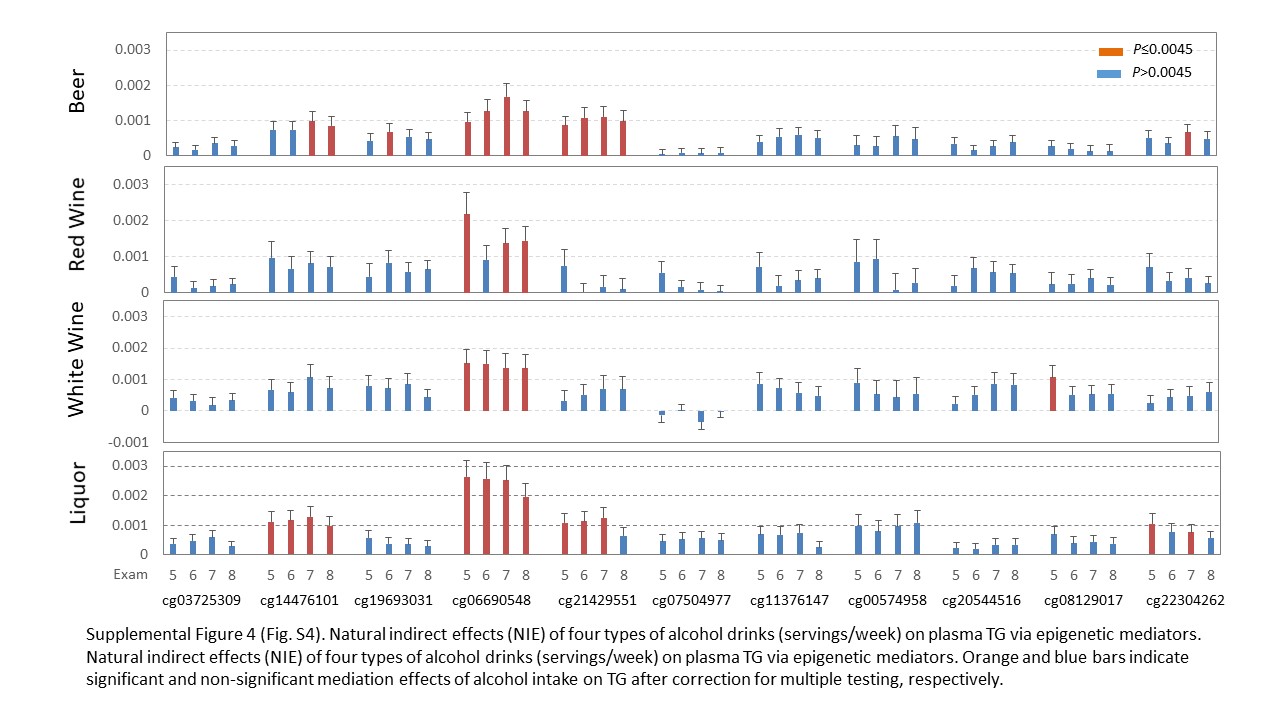

Supplement: Supplementary file 4 [file Image7.JPEG]

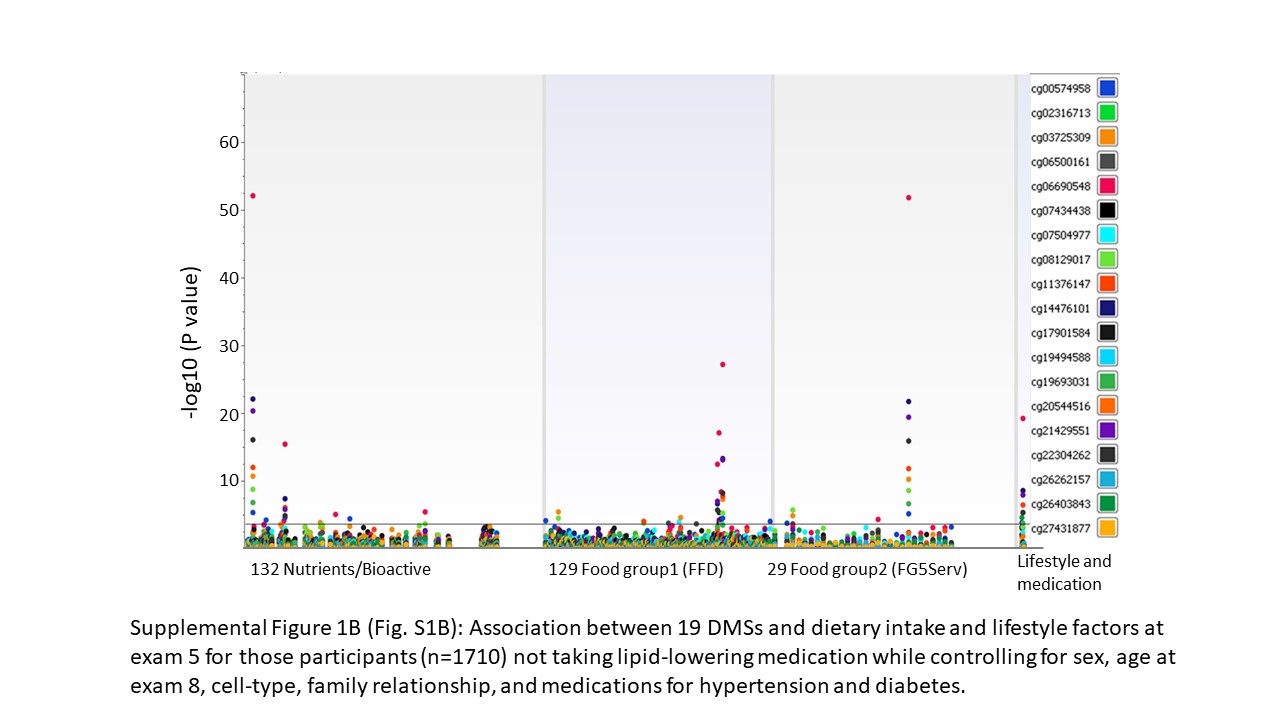

Supplement: Supplementary file 5 [file Image2.JPEG]

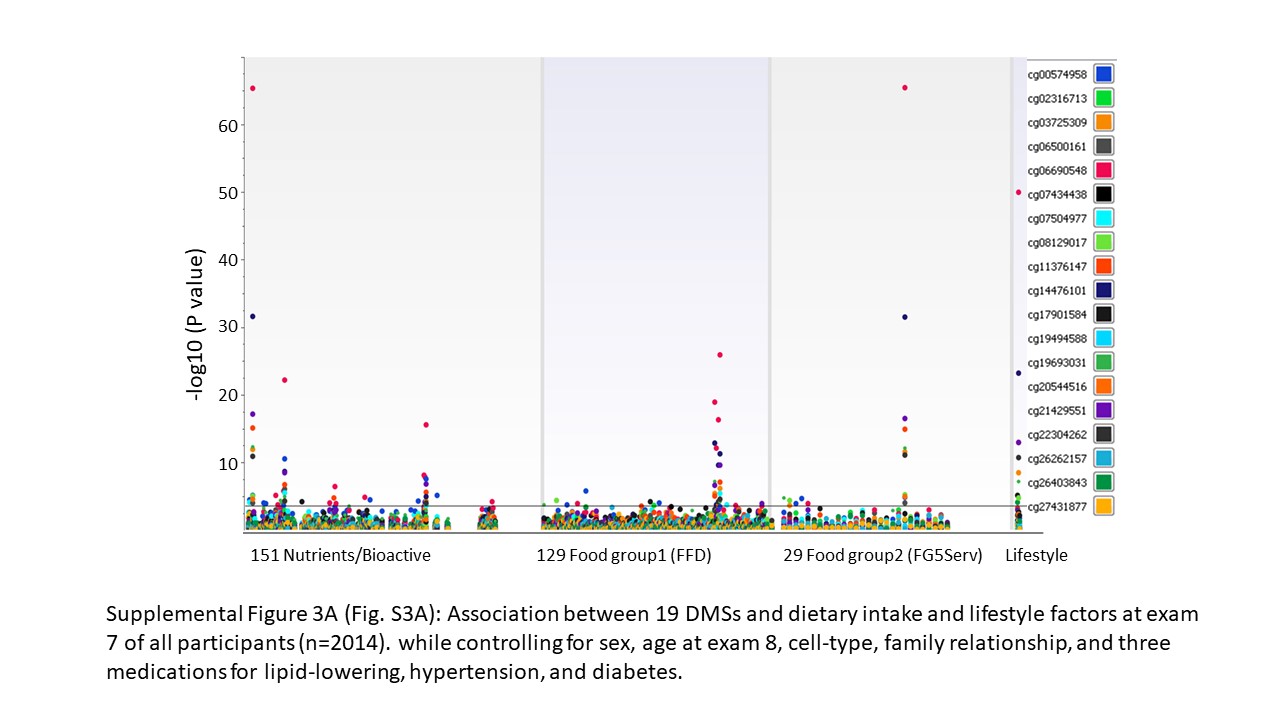

Supplement: Supplementary file 6 [file Image5.JPEG]

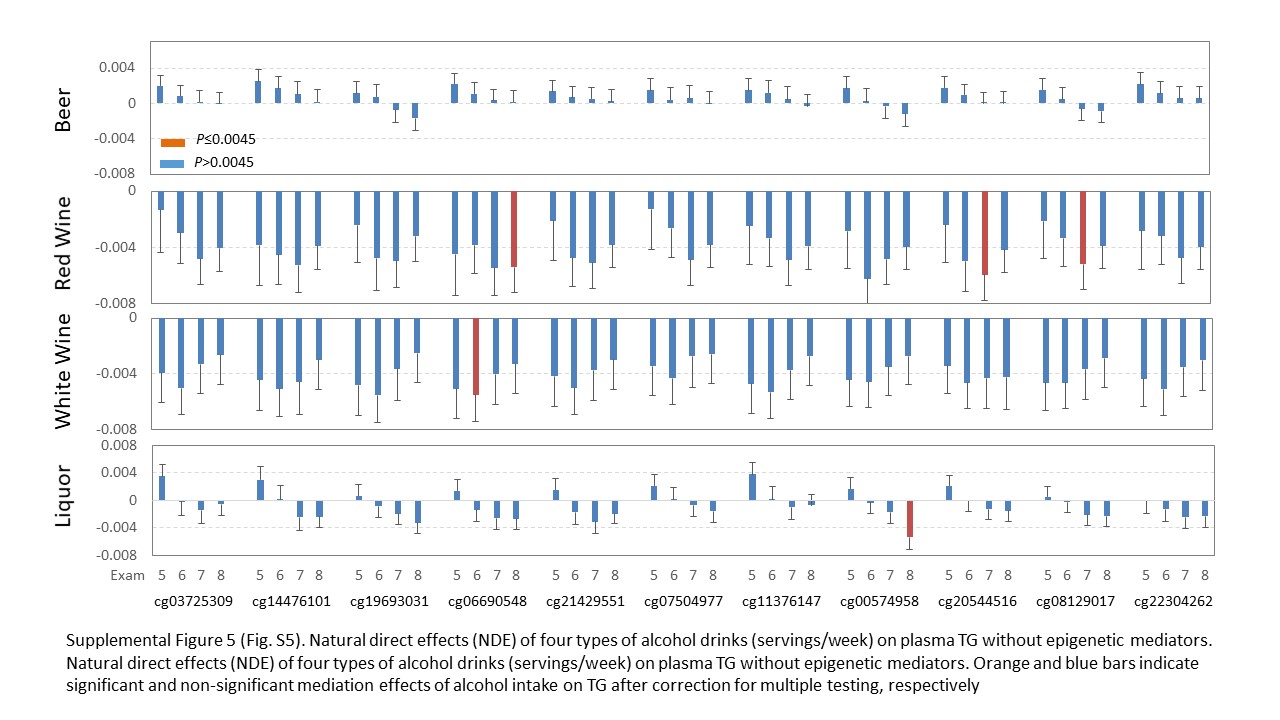

Supplement: Supplementary file 8 [file Image8.JPEG]

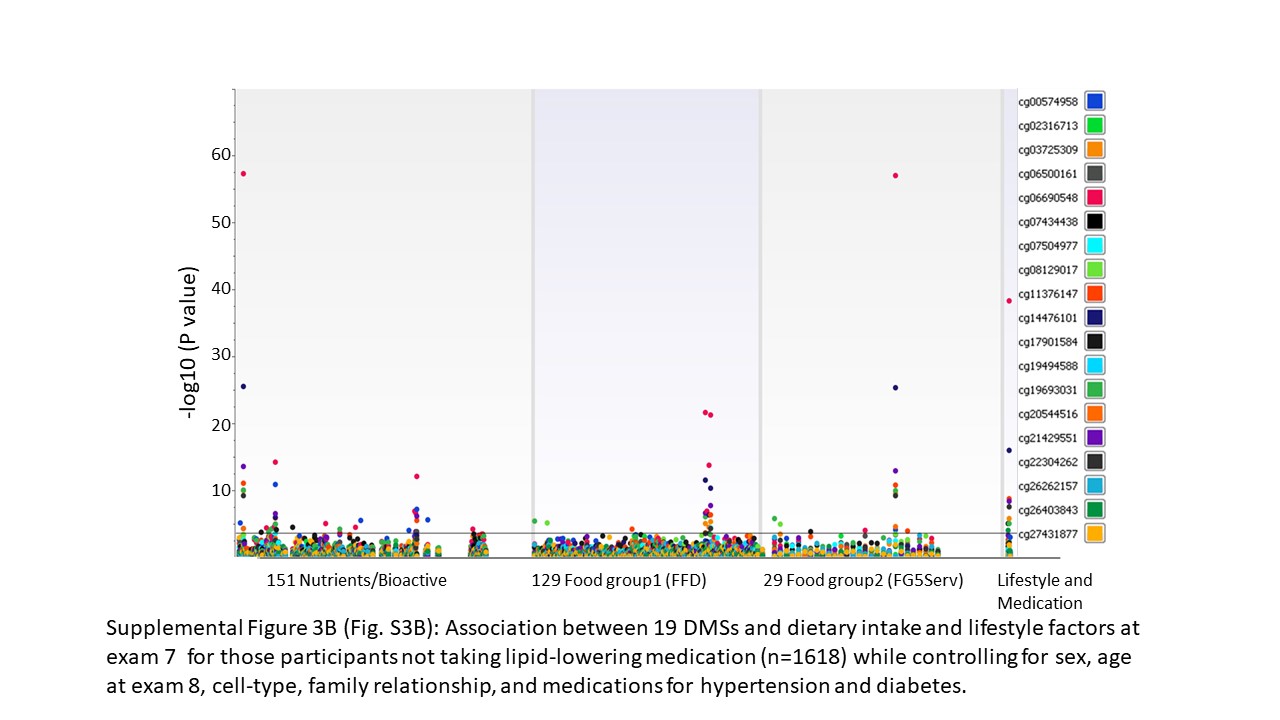

Supplement: Supplementary file 9 [file Image6.JPEG]
